# Sleep Quality during Exam Stress: The Role of Alcohol, Caffeine and Nicotine

**DOI:** 10.1371/journal.pone.0109490

**Published:** 2014-10-03

**Authors:** Matthias Zunhammer, Peter Eichhammer, Volker Busch

**Affiliations:** 1 Department of Experimental Psychology, University of Regensburg, Regensburg, Germany; 2 Clinic for Psychiatry and Psychotherapy, University of Regensburg, Regensburg, Germany; University of Utah, United States of America

## Abstract

Academic exam stress is known to compromise sleep quality and alter drug consumption in university students. Here we evaluated if sleeping problems and changes in legal drug consumption during exam stress are interrelated. We used the Pittsburgh Sleep Quality Index (PSQI) to survey sleep quality before, during, and after an academic exam period in 150 university students in a longitudinal questionnaire study. Self-reports of alcohol, caffeine, and nicotine consumption were obtained. The Perceived Stress Questionnaire (PSQ-20) was used as a measure of stress. Sleep quality and alcohol consumption significantly decreased, while perceived stress and caffeine consumption significantly increased during the exam period. No significant change in nicotine consumption was observed. In particular, students shortened their time in bed and showed symptoms of insomnia. Mixed model analysis indicated that sex, age, health status, as well as the amounts of alcohol and caffeine consumed had no significant influence on global sleep quality. The amount of nicotine consumed and perceived stress were identified as significant predictors of diminished sleep quality. Nicotine consumption had a small-to-very-small effect on sleep quality; perceived stress had a small-to-moderate effect. In conclusion, diminished sleep quality during exam periods was mainly predicted by perceived stress, while legal drug consumption played a minor role. Exam periods may pose an interesting model for the study of stress-induced sleeping problems and their mechanisms.

## Introduction

A substantial proportion of university students report poor sleep due to academic stress mid-term, with negative consequences for academic performance and well-being [1]. Perceived stress and somatic complaints have been shown to increase during exam periods [2] and so do sleeping problems. Ahrberg et al. (2012) [3] were the first to survey sleep-quality during exam stress with a longitudinal study design. They found that the sleep quality of medical students is reduced during exam periods and that poor sleep is associated with low academic performance. Astill et al. (2013) [4] examined the effects of exam stress on sleep quality in high-school students in a longitudinal actigraphy study. They showed that exam stress not only reduces sleep duration, but also the compensatory increase in sleep quality usually observed during periods of sleep withdrawal. These studies indicate that academic exam stress may pose an effective model for the study of stress-related sleeping problems with high external validity.

However, no longitudinal study has assessed the effects of exam stress on sleep quality in a general academic student sample so far. Moreover, a potential confounding factor has received insufficient attention: Students are known to alter their drug consumption during exam periods. Several studies showed that alcohol [5], [6], caffeine [6], [7], and nicotine [5]–[8] consumption change under exam stress. Since alcohol [9], [10], caffeine [11], and nicotine [12] are known for their detrimental effects on sleep quality, the reported decline in sleep quality during exam periods may partly be explained by changes in drug intake.

The objective of the present longitudinal questionnaire study was to assess how sleep quality, as well as alcohol, caffeine, and nicotine consumption change in in a general academic student sample before, during, and after an academic exam period. Further, we aimed at determining the degree of association between these phenomena by comparing the predictive value of legal drug consumption for sleep quality against a measure of perceived stress [16]. The global score of the Pittsburgh Sleep Quality Index (PSQI) [13] was selected as the main measure of sleep quality due to its good psychometric properties [14], [15] and for consistency with previous studies [1], [3].

## Methods

### Ethics Statement

Ethics approval (Ethikkommission der Universität Regensburg, Approval Number: 11-101-0239) and written informed consents were obtained. Data were collected and analyzed pseudonymously. Participants received a compensation of 8 Euros/hour.

### Participants

A naturalistic sample of 150 students was recruited across academic disciplines at two public Universities in Regensburg via bulletins, flyers, and personal appeal at lectures over two semesters. Past or present internal, neurological, hormonal, or psychiatric disorders were recorded. Data for the present study were collected in parallel to another survey – for further details regarding sample selection, study design, and time-course see: Zunhammer et al (2013) [2].

### Procedure

Written informed consent, medical history, and exam dates were obtained in a structured interview at a study inclusion visit. Afterwards, data were obtained using an online platform. Participants were asked to complete online-questionnaires before (Pre-Baseline), during (Exam Period), and after (Post-Baseline) an academic exam period. The Pre- and Post-Baseline time-points were scheduled within exam free periods, i.e. at a time-point where no exam was scheduled within the previous and next 30 days. The Exam Period time-point was scheduled within three days of the most “fearsome and/or distressing exam” of the current semester, as designated by the participant at study inclusion. The exam in question had to be prerequisite for graduation and/or contribute to the university degree. Participants were required to report any re-scheduling of exams. Exam dates were re-checked via the online platform at post-baseline. At each time-point, participants received a reminder by e-mail, or, if necessary, by telephone.

Participants were asked to complete an online version of the German PSQI [13]. The PSQI is an established questionnaire for the measurement of sleep quality [14], [15]. It consists of 9 self-report items and an additional item asking for observations made by room/bedmates. The additional item was not assessed in the present study. Higher PSQI-scores denote decreased sleep quality. Poor sleep has been defined as a PSQI global score of > = 5 [13]. This cutoff has been shown to have high specificity and sensitivity for distinguishing insomnia patients and healthy controls [13], [15]. Instructions and wording of the PSQI online form were identical to the original paper version, but missing items were precluded by forced-choice settings. The time frame of assessment was the past month, as in the original version [13].

The Regensburg Insomnia Scale (RIS) is a short rating scale for the assessment of psychophysiological insomnia [17]. It consists of 10 items querying typical insomnia symptoms. Higher scores denote increased symptom severity. Symptom severities are rated on five-point Likert scales. The RIS was included as an additional measure of sleep quality in the present study with a focus on insomnia. The time frame of assessment of the RIS was the past month.

The amounts of alcohol (in drinks), caffeine (in units), and nicotine (in cigarettes) consumed within the past week were surveyed in a separate online form. An alcoholic drink was defined as 150 ml of wine, 333 ml of beer, or 40 ml of liquor (∼ 13 g of ethanol). An unit of caffeine was defined as 200 ml of coffee or tea, or 500 ml of caffeinated soft drinks (∼ 50 to 100 g of caffeine). Again, missing items were precluded by forced-choice settings.

For the present and the parallel study [2], acute infections, injuries, and exacerbations of pre-existing conditions were surveyed using an online form asking for health status and a description of current symptoms. The obtained reports of current health status were categorized into “ill/injured” or “healthy” by three investigators independently. In case of doubt participants were classified as “ill/injured”.

The Perceived Stress Questionnaire (PSQ-20) [16] was obtained as a self-report measure of stress for both the present and the parallel study [2]. The PSQ-20 consists of 20 negatively and positively worded items, asking for typical indicators of stress, e.g. “Your problems seem to be piling up”, “You have trouble relaxing”, or “You have enough time for yourself”. Items are rated on a 4-point likert scale (1 “almost never”, 2 “somtimes”, 3 “often”, 4 “usually”). The time frame of assessment chosen for the PSQ-20 was the past week. The PSQ-20 total score was used as an additional predictor of sleep quality and used to verify that the exam period was perceived as stressful.

### Statistics

Statistical tests were performed with SPSS 21.0.0.0 for Mac OS at a two-tailed *α* <0.05. Means are given ± standard deviation if not denoted otherwise. Friedman’s test statistic (X^2^_F_) was used in combination with Sidak-corrected post-hoc tests to determine changes in PSQI and drug intake over time. In addition, mixed modeling was employed to estimate to what degree legal drugs and perceived stress account for the effects of Exam Period on PSQI global score. SPSS’s MIXED function with maximum likelihood (ML) estimation was used to test the effects of the fixed factors time (levels: Pre-Baseline, Exam Period and Post-Baseline), sex (levels: male, female), health status (levels: ill/injured, healthy), as well as the effects of the fixed co-factors age (in years), alcohol (in drinks/week), caffeine (in units/week), nicotine (in cigarettes/week), and perceived stress (PSQ-total score) on PSQI global score. All co-factors were mean-centered before mixed model analysis. A random, by-subject intercept was included in the model to account for within-subject dependencies introduced by the repeated-measures design. Marginal and conditional R^2^ values were used to describe model fit [18]. Cohen’s *f^2^* [19] was calculated as a measure of effect size according to Selya et al. (2012) [20].

## Results

The complete dataset of the present study (including data from the parallel study [2]) is available as an online supplement (see: Data S1).

### Sample

**Figure 1:**
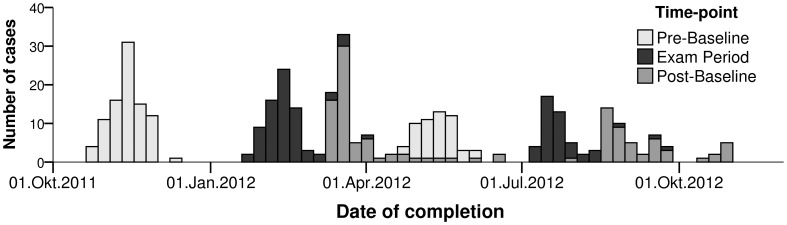
Histogram of time-points of acquisition. Participants were recruited in winter semester 2011 and summer semester 2012. Lecture periods within these semesters were 17. Okt.2011-11. Feb.2012 and 16. Apr.2012–21. Jul.2012.

Analysis included 142 participants (71 female), of which 106 contributed three, 24 two, and 12 one valid session(s). Figure 1 shows a histogram of the time-points of data-collection. Eight (2 female) participants were excluded from the study. The reasons for study exclusion were severe internal diseases (n = 3), lost to follow up (n = 3), university drop out (n = 1), and voluntary drop out (n = 1). The sample was identical with the sample of the main study [2], except for three additional cases excluded due to ambiguous bed−/wake-times given in the PSQI form.

Sixteen (8 female) participants had a history of major internal or psychiatric disorders or stable chronic disorders. These were: migraine (n = 5), chronic back pain (n = 3), chronic joint pain (n = 2), depression (n = 2), tinnitus, urticaria, orofacial pain, and social anxiety (n = 1, each). Participants were from the fields of humanities (27.5%), psychology/medicine (26.8%), natural sciences, engineering, mathematics, or informatics (22.5%), economics (5.6%), and interdisciplinary/others (17.6%).

### Sleep quality

Descriptive and statistical results for all scales of the PSQI are given in Table 1. Friedman’s test (Table 1) indicated that PSQI global score was significantly elevated at Pre-Baseline and Exam Period compared to Post-Baseline (see: Table 1). All sub-scales of the PSQI contributed to the decline in sleep quality at Exam Period, except for the “sleep disturbance” and “use of hypnotics” sub-scales (see: Table1). PSQI global scores, pooled across time-points, did not differ significantly between the sexes (males-females: −0.03±2.15 units, n = 142, U = 2468.0, p = .832) and showed no significant association with age (n = 142, Kendall’s Tau-b rank correlation coefficient = −0.03, p = .580). The RIS supplemented the results of the PSQI by indicating a significant increase in insomnia symptoms during the Exam Period (see: Table 1) compared to Pre-and Post-Baseline.

**Table 1:** Sleep quality and legal drug consumption before, during and after an exam period.

	Pre-Baseline	ExamPeriod	Post-Baseline	Friedman’s Statistic	
n	n = 139	n = 122	n = 117	(df = 2, n = 106)	
Variable	Mean ± SD			X^2^_F_,	Post-Hoc
**Pittsburgh Sleep Quality Index (PSQI):**					
Bedtime (Daytime ± h)	23∶47±1.11	23∶50±1.32	00∶01±1.70	–	–
Waketime (Daytime ± h)	08∶06±1.25	07∶58±1.42	08∶40±2.66	–	–
Time in Bed (h)	8.31±0.96	8.14±1.11	8.65±1.22	12.54**	B**
Sleep Onset Latency (min)	19.6±23.0	24.3±21.8	17.8±18.5	20.50***	B**
% of Cases with Sleep Onset Latency ≥30 min	22.3%	34.4%	18.8%	–	–
Reported Sleep Time (h)	7.35±1.01	7.10±1.04	7.84±1.21	17.53***	B**
Sleep Efficiency Sub-Scale	0.42±0.68	0.57±0.96	0.37±0.85	5.42^n.s^	–
Sleep Efficiency in %	88.7±0.1%	87.9±0.1%	90.6±0.1%	13.99**	B*, C*
Subjective Sleep Quality	0.43±0.68	0.57±0.96	0.37±0.85	40.75***	A**, B***
% of Cases with Very Good/Fairly Good/FairlyBad/Very Bad Sleep Quality	27.3/61.2/10.1/1.4%	7.4/66.4/23.0/3.3%	31.6/62.4/5.1/0.9%	–	–
Hypnotics use:	0.02±0.15	0.04±0.27	0.02±0.18	– not enough cases	
Daytime Dysfunction/Sleepiness Score	1.03±0.75	1.28±0.75	0.81±0.73	28.19 ***	B ***, C *
Sleep Disturbance Sub-Scale	0.99±0.32	0.97±0.34	0.90±0.40	3.85	–
PSQI Global Score	4.95±2.53	6.25±2.82	4.10±2.39	47.43 ***	A ***, B ***, C *
% of Cases with PSQI >5	34.5%	53.3%	21.4%	–	–
**Additional Sleep Scales:**					
Regensburg Insomnia Scale (RIS)	7.12±4.93	8.79±5.51	6.24±3.96	29.06***	A**, B***
**Legal Drug Consumption and Current Health Status (Self-report):**					
Caffeine (Cups)	4.76±5.67	7.10±9.51	4.56±5.52	25.88 ***	A **, B **
Nicotine (Cigarettes)	7.24±25.54	7.19±29.12	5.39±20.75	0.45 ^n.s.^	–
Alcohol (drinks)	5.94±6.31	3.42±4.42	6.29±7.88	19.16 ***	A **, B *
% of Cases with Acute Infections, Injuries,or Exacerbations of Pre-Existing Conditions	18.7	8.2	18.8	–	–
**Perceived Stress**					
Perceived Stress Scale (PSQ-20)	33.68±17.62	54.06±19.34	29.62±18.24	98.34***	A***, B***, C*

Higher PSQI scores denote lower sleep quality. Friedman’s test statistic (X^2^_F_) was used to determine changes in PSQI and drug intake over time.

Abbreviations: Post-hoc differences are denoted as “A”: between Pre-Baseline and Exam Period, “B”: between Exam Period and Post-Baseline, and “C”: between Pre- and Post-Baseline. n.s.: not significant. * p<0.05, ** p<0.01, *** p<0.001.

### Legal drug consumption and health status

Descriptive and statistical results for drug consumption habits are also given in Table 1. The majority of participants (94.4%) reported the consumption of alcoholic beverages; 33.1% of participants reported risky drinking at least at one time-point. Risky drinking was defined as a weekly alcohol consumption equal or greater 168 g of pure ethanol for men and 84 g for women, or less than two days of abstinence/week according to guidelines by the German Federal Center for Health Education [21]. The consumption of caffeinated beverages was reported by 111 (78.2%) participants. Smoking was reported by 38 participants (26.8%), but only 13 participants (9.1%) smoked regularly (> = 3.5 days/ week). At mean, regular smokers smoked 64.8±52.6 and occasional smokers 4.98±6.17 cigarettes/week.

Male participants reported to consume significantly higher amounts of alcohol (males-females: +3.65±4.77 drinks/week, n = 141, U = 3353.5, p<.001) and nicotine (males-females: +9.74±19.23 cigarettes/week, n = 142, U = 3209.0, p<.001), according to Mann-Whitney-U Tests (data pooled across time-points). Caffeine consumption (males-females: +0.47±6.34 cups of coffee/week, n = 142, U = 2504.0, p = .946) did not differ significantly between the sexes. Alcohol and caffeine consumption were not found to be associated with age across time-points, but the number of cigarettes consumed significantly correlated with age (n = 142, Kendall’s Tau-b rank correlation coefficient = 0.16, p = .020). The prevalence of acute infections, injuries, or exacerbations of pre-existing conditions is given in Table 1.

### Exploring predictors of sleep quality

**Figure 2:**
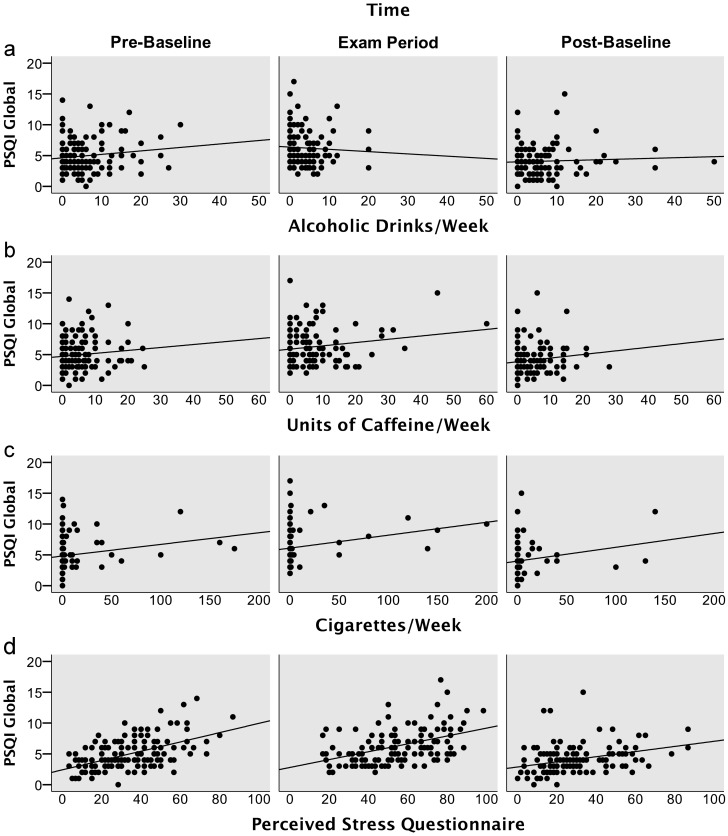
PSQI global scores plotted against consumed amounts of alcohol (2a), caffeine (2b), and nicotine (2c), as well as PSQI global scores plotted against perceived stress (2d) within the last week. Perceived stress and the amount of nicotine consumed were identified as significant predictors of PSQI global score. Alcohol and caffeine did not predict PSQI global score significantly.

The effects of time, sex, age, health status, alcohol (Figure 2a), caffeine (Figure 2b), nicotine (Figure 2c), and perceived stress (Figure 2d) on PSQI global score were tested using a mixed model. Parameter estimates and statistical tests are given in Table 2. Perceived stress was the best predictor of global sleep quality showing a small-to-moderate effect size [19]. Increases in perceived stress were associated with decreased sleep quality. The weekly amount of cigarettes smoked significantly predicted sleep quality with a small effect size. Increased cigarette consumption was associated with decreased sleep quality. Sex, age, current health status, alcohol, and caffeine did not predict significant proportions of variance in PSQI global score. After accounting for age, sex, health status, legal drugs, and perceived stress, PSQI global score was still significantly elevated at Pre-Baseline and Exam Period, compared to Post-Baseline (see: Table 2). However, the effect size was small, since the proportion of total variance explained by factors time and perceived stress overlapped: Both factors shared 13.9% of total variance. The unique proportion of variance explained by time was 2.41%, the unique proportion of variance explained by perceived stress 8.55%.

**Table 2:** Mixed model results: predictors of sleep quality.

Model Parameter	Units	*β*	95% CI (lower)	95% CI (upper)	*t*	*p*	Cohen’s *f^2^*
**Intercept**	Mean PSQI Score at Post-Baseline	4.50	3.76	5.24	11.95	<.001	
**Pre-Baseline**	Dummy coded contrast with Post-Baseline	0.60	0.18	1.02	2.82	**.005**	0.040†
**Exam Period**	Dummy coded contrast with Post-Baseline	0.62	0.08	1.16	2.26	**.025**	0.040†
**Sex**	Female	−0.20	−0.87	0.46	−0.60	.548	0.001
**Age**	Years	−0.034	−0.171	0.102	−0.50	.620	0.000
**Health Status**	Ill/injured compared to healthy	−0.36	0.24	−0.96	−1.19	.237	0.010
**Alcohol**	Drinks/week	−0.030	−0.070	0.010	−1.47	.142	0.021
**Caffeine**	Units/week	0.026	−0.011	0.063	1.39	.165	0.005
**Nicotine**	Cigarettes/week	0.020	0.007	0.032	3.16	**.002**	0.018
**Perceived Stress**	Score (mean centered)	0.054	0.042	0.067	8.47	**<.001**	0.140

Estimated unstandardized coefficients (***β***) of the mixed model, with corresponding t-tests against the null-hypothesis of no effect. The mixed model included a random intercept term for each participant. Positive beta values indicate an increase in PSQI score and therefore a decrease in sleep quality. All co-variates were mean-centered. †Value represents effect size of factor time.

### Additional analyses

The mixed model accounted for 64.3% of total variance in PSQI global score (conditional R^2^) [18]. The fixed factors alone explained 31.0% of total variance in PSQI global score (marginal R^2^) [18]. Akaike’s information criterion corrected for finite sample sizes (AICc) decreased by 67.2 points when including the random intercept term, which additionally confirmed that the by-subject intercept was crucial for model fit. An analysis of predicted values revealed that the model shown in Table 2 tended to underestimate high PSQI global scores. A residual analysis further revealed a tendency for increased variance at high PSQI total scores. Excluding PSQI scores > = 9 resolved these problems and led to identical results in respect to the criterion of significance. Similarly, excluding cases reporting more than 10 drinks/week, 10 units of caffeine/week did not affect results. The effect of nicotine vanished when cases smoking more than 50 cigarettes/week were excluded. Obviously this was due to the low number of smokers in the sample.

## Discussion

### Effects of an exam period on sleep

University students from various academic disciplines reported decreases in sleep quality during an exam period compared to two exam-free baselines (see: Table1). PSQI global score increased by a mean of 1.7 points and the proportion of poor sleepers in the sample increased by 25.4%, to 53.3% (see: Table1). These figures illustrate that a substantial proportion of university students report sleeping problems within the month before an important exam. Determining the consequences of sleeping problems during exam stress for academic performance was beyond the scope of the present study. However, the observed effect sizes are similar to estimates from a study with medical students, which could show that sleeping problems during the exam period are pronounced in students with low performance [3].

In particular, total sleep time, subjective sleep quality, and daytime sleepiness have been associated with academic performance [22], [23], although parameters like morningness/eveningness and early/late bedtimes have also been shown to play a role [24], [25]. In the present study we found that students reported to spend significantly less time in bed during the exam period, 0.5 hours less on average (see: Table 1). This reduction suggests that students alter their sleeping schedule while studying for exams. Moreover, participants reported significant decreases in total sleep time, sleep efficiency, and subjective sleep quality, plus increases in sleep onset latency and daytime sleepiness (see: Table 1). These results indicate that students suffer from symptoms of insomnia during the exam period, a notion confirmed by significant increases in RIS score (see: Table 1). The sleep disturbance sub-scale of the PSQI, which covers disrupted sleep due to bodily symptoms and sleep terrors, was not significantly affected by the Exam Period (see: Table 1). Use of hypnotics did not change significantly either, however, only 5 of 142 participants reported to use hypnotics.

The effects of Exam Period were more pronounced when compared to Post-, rather than Pre-Baseline. Further, sleep efficiency, daytime sleepiness, and PSQI global score significantly differed between Pre- and Post-Baselines, pointing towards better sleep quality at Post-Baseline. These effects might be explained by the fact that Pre-Baseline measurements were predominantly obtained during lecture terms, while Post-Baselines were predominantly obtained during semester breaks (see: Figure 1). Sample attrition in combination with a higher drop-out rate amongst poor sleepers may pose an alternative explanation for these differences, as 15% of the participants partaking at Pre-Baseline dropped out until Post-Baseline.

### Effects of exam period on parameters of legal drug use

We could replicate previous studies showing that alcohol consumption decreases [5], [6], but caffeine consumption increases [6], [7] during exam periods. Contrary to previous studies [5]–[8] we could not find significant changes in nicotine consumption during the exam period–neither within the full sample (see: Table 1), nor within the smokers. Moreover, we replicated the finding that male students consume more alcohol and nicotine than female [5].

### Associations between sleep, legal drugs and perceived stress

We performed a mixed model analysis to explore how demographic variables, health status, legal drug consumption, and perceived stress affect sleep quality over the study course. A random intercept was used to account for within-subject dependencies introduced by our repeated-measures design. Conditional and marginal R^2^ confirmed that the by-subject random intercept term accounted for a substantial amount of variance in PSQI global score. The fixed factors age, sex, and current health status had no significant effect on global sleep quality. Despite significant changes in alcohol and caffeine consumption during exam period, the consumed amounts of these drugs were not significantly associated with sleep quality (see: Figures 2a and b). These findings are in accord with a previous study, which indicated that caffeine intake increases during exam periods but has no relevant effect on sleep quality [4]. However, the present results are at odds with another study finding that the amount of alcohol consumed is a predictor of several parameters of sleep behaviour [23]. Nicotine was the only legal drug which showed a significant relationship with sleep quality across time-points. Smoking a pack of 20 cigarettes/week was associated with an increase in PSQI global score by 0.40 (95% CI [0.14; 0.64]) points. The standardized effect of nicotine was small and only 38 participants in the sample reported smoking at all (see: Figure 2c). Therefore, these results must be taken with a grain of salt. Nevertheless, the results are in line with a large multicenter study showing that smokers experience more sleeping problems than non-smokers [10].

In contrast to legal drug use, perceived stress was identified as a significant predictor of sleep quality across time-points (see: Figure 2d). In fact, perceived stress was the best predictor in the model, with a small-to-moderate effect size. This substantiates the notion that stress-related mechanisms underlie the observed sleeping problems of university students during exam periods. Our results tally with findings of a large cross-sectional study, which investigated college students mid-term [1]. Lund et al. (2010), found that “tension” as measured by a sub-scale of the Profile of Mood States (POMS) and a second stress scale accounted for most of the explained variance in sleep quality, while alcohol and caffeine consumption were predictors of minor importance [1]. Our present study provides a further example for the effects of psychosocial stress on sleep. Likewise, work stress [26] and interpersonal stress [27] have been identified as predictors of low sleep quality. Cognitive mechanisms behind the observed sleeping problems during exam stress may include hyperarousal and rumination, i.e. excessive thinking about the current stressor [26], [27]. Potential neurophysiological mechanisms of relevance include changes in HPA-axis function [28] and autonomic nervous system activity [29]. Further studies on stress-induced insomnia might help to identify its underlying mechanisms and to optimize therapeutic options for students suffering from impaired academic performance due to excessive sleep loss.

### Limitations

Like all questionnaire studies, our study is vulnerable to response bias. The sample was obtained from two large Universities located in Regensburg, which both draw their students mainly from rural Eastern Bavaria [30]. Therefore, the present results may not generalize to students in other countries or regions. Further, the sleep quality in our naturalistic student sample was comparable to healthy samples [14], [15]. The present data therefore mainly reflect the situation in healthy students. Individuals with pre-existing sleeping disorders, or a vulnerability for drug addiction may show different behavior during exam stress. We want to emphasize that, except for the factor “time”, no predictor variable was under experimental control. A causal relationship between predictors and PSQI score can therefore not be inferred. Third-variables such as stress vulnerability might underlie the observed associations between PSQI-score, nicotine consumption, and perceived stress. Of note, the time frame of the PSQI differed from the time frames of the predictor variables used in mixed modeling. The PSQI inquired sleep quality within the last month. Health status, legal drug consumption, and perceived stress were surveyed for the past week. These differences in time frame may have reduced the associations between sleep quality and the predictor variables.

### Conclusion

The present study provides estimates of the effects of exam stress on sleep quality and legal drug consumption in university students. Students were found to report a general reduction in sleep quality during exam periods. In particular, students reported to shorten their time-in-bed and to suffer increased symptoms of insomnia during an exam period. Alcohol and caffeine consumption changed under exam stress, too, but were not found to significantly predict sleep quality. In contrast, nicotine consumption did not change but was found to significantly predict decreases in sleep quality. Perceived stress was the best predictor of sleep quality evaluated. These results indicate that sleep quality in university students decreases during academic exam periods and that these decreases are stress-related. The present results indicate that exam periods are a promising model setting for the study of stress-related insomnia, its consequences, and potential remedies. Future studies are desirable to identify the mechanisms behind sleeping problems during exam stress.

## Supporting Information

Data S1 **Original data in SPSS (.sav) and comma delimited text (.csv) format.**
(ZIP)
